# P-808. Enterococcal Bloodstream Infection and Infective Endocarditis in a Population-Based Cohort: An Expanded Rochester Epidemiology Project Investigation

**DOI:** 10.1093/ofid/ofae631.1000

**Published:** 2025-01-29

**Authors:** David Bayless, Larry M Baddour, Brian Lahr, Grace Hagan, Jenny Cao, Daniel C DeSimone

**Affiliations:** Mayo Clinic College of Medicine & Science, Rochester, Minnesota; Mayo Clinic College of Medicine, Rochester, MN; Mayo Clinic, Rochester, Minnesota; Mayo Clinic College of Medicine & Science, Rochester, Minnesota; Mayo Clinic College of Medicine & Science, Rochester, Minnesota; Mayo Clinic, Rochester, Minnesota

## Abstract

**Background:**

The Rochester Epidemiology Project (REP) has been used to examine numerous infectious and non-infectious entities in a population-based approach in Olmsted County, Minnesota. To further investigate patterns of enterococcal bloodstream infection (BSI), we used the Expanded Rochester Epidemiology Project (E-REP) to include additional surrounding counties to conduct a retrospective population-based study of adult patients who developed enterococcal BSI, with or without infective endocarditis (IE).

**Figure:**
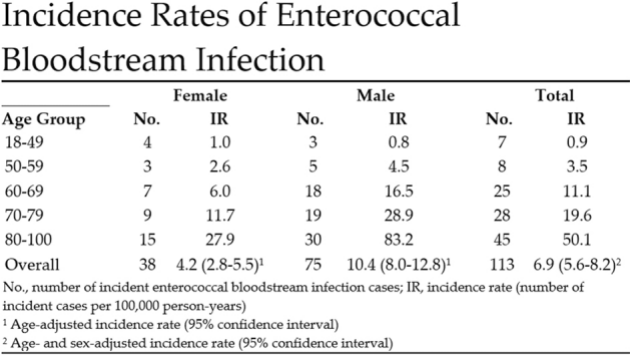

**Methods:**

The E-REP population under study included eight counties in southeastern Minnesota (including Olmsted County), representing a total population of over 250,000 individuals. Adults (age ≥ 18 years) diagnosed with an initial episode of enterococcal BSI with or without enterococcal IE between January 1, 2018 and December 31, 2022 were included; patients with either polymicrobial BSI with enterococci or a previous bout of enterococcal BSI before the study period were excluded. Cases of enterococcal BSI and IE were identified using ICD-10 codes and underwent complete medical record review. Patient characteristics, microbiology, antibiotic regimens, infectious complications, and outcomes were collected.

**Figure:**
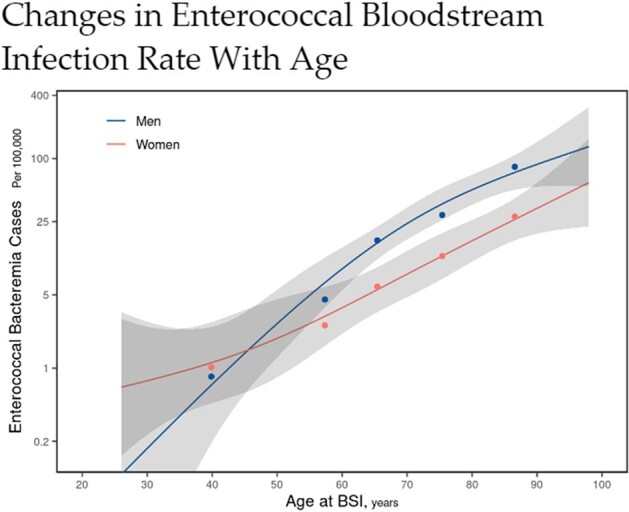

**Results:**

A total of 113 cases of enterococcal BSI were identified over the five-year study period. The age- and sex-adjusted incidence rate of enterococcal BSI was 6.9 per 100,000 person-years, with a significantly higher rate among males than females (Figure 1). BSI incidence rate varied by age, with patients 80 years or older having the highest incidence rate (Figures 1 and 2). Twenty-two cases of enterococcal IE were identified, yielding an overall IE prevalence of 20.8% (Figure 3). The cumulative mortality rate at 12 months was high at 41.7% (Figure 3). Five patients (4.4%) had a relapse of enterococcal BSI, defined as recurrence of BSI after negative follow-up blood cultures and within 12 weeks of the initial BSI.

**Figure:**
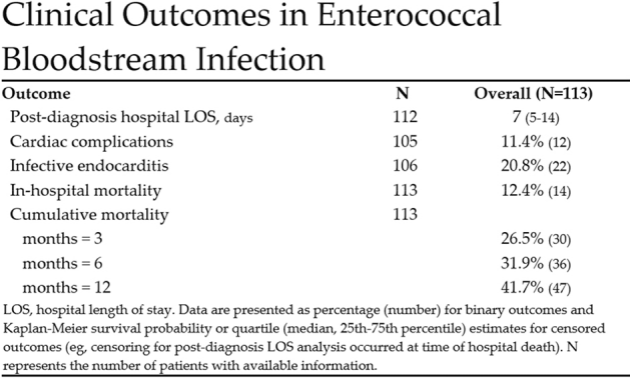

**Conclusion:**

This is the first population-based investigation that has focused on the epidemiology and clinical profile of patients with enterococcal BSI and complicating IE in a US population. This included an IE prevalence of 20.8%, which should be considered when evaluating and managing patients with enterococcal BSI.

**Disclosures:**

**Larry M. Baddour, MD**, UpToDate, Inc.: Royalty payments (authorship duties).

